# Draft Genome Sequence of Escherichia coli Strain Tj, Isolated from the Varzob River in Tajikistan

**DOI:** 10.1128/MRA.00867-20

**Published:** 2020-10-08

**Authors:** Munavvara Dzhuraeva, Mehrangez Shokirova, Ani Azaryan, Hovik Panosyan, Khursheda Bobodzhanova, Nils-Kåre Birkeland

**Affiliations:** a Center of Biotechnology, Tajik National University, Dushanbe, Tajikistan; b Department of Biochemistry, Microbiology, and Biotechnology, Yerevan State University, Yerevan, Armenia; c Department of Biological Sciences, University of Bergen, Bergen, Norway; University of Maryland School of Medicine

## Abstract

The 4.6-Mbp draft genome sequence of Escherichia coli strain Tj, isolated from the Varzob River in Tajikistan, is presented. This strain possesses four prophage elements related to *Shigella* phage SfV, E. coli O157:H7-specific phage ϕV10, lambdoid phage HK225, and coliphage Ayreon. It contains a gene encoding a hemolysin E toxin.

## ANNOUNCEMENT

**FIG 1:**
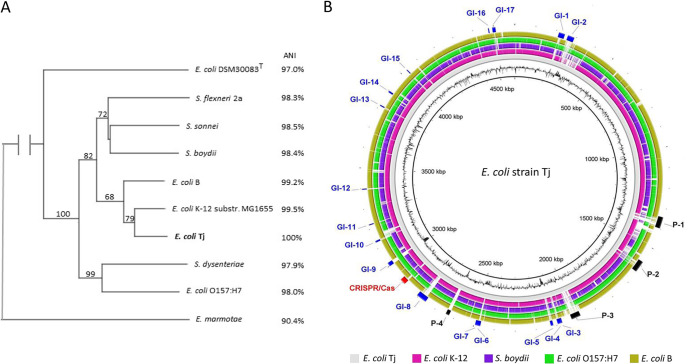
Genome-based phylogenetic affiliation of strain Tj with representative E. coli strains and *Shigella* spp., represented as a phylogenetic tree (A), and circular representation of the E. coli Tj genome as a reference, compared with representative E. coli strains and *Shigella* spp. (B), using the BLAST Ring Image Generator (BRIG) (10). The tree was inferred with FastME v. 2.1.6.1 (11) from Genome Blast Distance Phylogeny (GBDP) distances calculated from genome sequences using the TYGS server (https://tygs.dsmz.de) (12) and rooted with Escherichia marmotae as an outgroup. The branch lengths are scaled in terms of the GBDP distance formula d5. The numbers above the branches are GBDP pseudobootstrap support values of ≥68% from 100 replications. The tree was rooted at the midpoint (13). Before being used as a reference genome for the BRIG, the Tj contigs were ordered using Mauve (14) with the E. coli K-12 genome as the template and merged. Ring color codes are indicated below the rings. The black (innermost) ring indicates the GC content of strain Tj. Selected genomic features of strain Tj are indicated as GIs, prophage elements (P), and a CRISPR/Cas element. Genome sequence accession numbers are as follows: GCA_007049865.1 (E. coli Tj), NC_000913.3 (E. coli strain K-12 substrain MG1655), NC_002695.2 (E. coli O157:H7 strain Sakai), NZ_CP014268.2 (E. coli B), NZ_CP025979.1 (E. marmotae), NZ_CP026731.1 (Shigella boydii), NZ_CP026774.1 (Shigella dysenteriae), NZ_CP026788.1 (Shigella flexneri 2a), CP026802.1 (Shigella sonnei), and NZ_CP033092 (E. coli DSM 30083^T^).

Escherichia coli (Migula 1895) Castellani and Chalmers 1919 is a Gram-negative, facultatively anaerobic, enteric bacterium that inhabits the intestines of warm-blooded animals and humans and frequently contaminates environmental waters and food. E. coli strain Tj was isolated from the Varzob River in Tajikistan, during a laboratory course at the Center of Biotechnology of the Tajik National University, on Endo LES agar plates at 44°C as a dark-red colony and confirmed as E. coli by its API 20E profile (5-0-4-4-5-5-2). The 16S rRNA gene sequence, obtained by PCR amplification and Sanger sequencing as described elsewhere (1), shared up to 99.86% sequence identity with E. coli strains in BLASTn searches. For genome sequencing, the strain was cultivated in LB overnight at 37°C with shaking. DNA was extracted using the GenElute bacterial genomic DNA kit (Sigma-Aldrich). The genome was sequenced at Eurofins Genomics using a NEBNext Ultra II DNA library preparation kit and Illumina HiSeq 2500 paired-end sequencing technology with a read length of 2 × 150 bp, yielding 6,416,190 reads and 1,924,857,000 sequenced bases. Reads with a maximum of 7 bases with a Phred score below 28 were initially discarded, and additional quality control procedures were performed using the Trim Reads tool in the CLC Genomics Workbench v. 8.5.1. Unless otherwise stated, all software was used with default values. Assembly was performed using the CLC *de novo* assembly tool, resulting in 4,627,784 bp of unique sequence data distributed into 96 contigs with an *N*_50_ value of 111,402 bp, coverage of 417×, and GC content of 50.8%. The draft genome was annotated using the NCBI Prokaryotic Genome Annotation Pipeline (PGAP) (https://www.ncbi.nlm.nih.gov/genome/annotation_prok). Genome completeness was estimated as 99.96% by CheckM v. 1.0.18 (2). A phylogenomic analysis, including a selection of E. coli strains and *Shigella* species, revealed clustering within the E. coli*/Shigella* clade, with pairwise average nucleotide identity (ANI) values of ≥97.0% (Fig. 1A). Only one enterobacterial toxin-encoding gene, a pore-forming hemolysin E homolog, was detected in strain Tj in a search of the 2016 release of the Virulence Factor Database (http://www.mgc.ac.cn/VFs) (3) using the VFanalyzer tool. However, many genomic islands (GIs), four prophage elements (P), and one CRISPR/Cas element were found (Fig. 1B) using IslandViewer 4 (4), Prophage Hunter (5), and CRISPRCasFinder (6), respectively. The prophage elements were related to E. coli phage Ayreon (P-1) (7), the serotype-converting temperate phage SfV from Shigella flexneri (P-2) (8), lambdoid coliphage HK225 (GenBank accession number NC_019717), and phage ϕV10, which specifically infects E. coli O157:H7 (P-4) (9). Most of the GIs encoded mobile elements and hypothetical genes. GI-1, GI-9, and GI-10 encoded type III and type VI secretion systems and a general secretion pathway, respectively. The reported data will be useful for future understanding of the genetic diversity and virulence potential of E. coli in Central Asia and the distribution, evolution, and dissemination of coliphages.

### Data availability.

The partial 16S rRNA gene and whole-genome shotgun sequences of E. coli strain Tj have been deposited in DDBJ/ENA/GenBank under accession numbers MT920313 and GCA_007049865.1, respectively. The associated BioProject, SRA, and BioSample accession numbers are PRJNA506592, SRR12336902, and SAMN10464432, respectively.
